# “It's no good but at least I've always got it round my neck”: A postphenomenological analysis of reassurance in assistive technology use by older people

**DOI:** 10.1016/j.socscimed.2021.114553

**Published:** 2022-01

**Authors:** Jennifer Lynch, Gemma Hughes, Chrysanthi Papoutsi, Joseph Wherton, Christine A'Court

**Affiliations:** a Centre for Research in Public Health and Community Care, University of Hertfordshire and National Institute for Health Research Applied Research Collaboration East of England, United Kingdom; b Nuffield Department of Primary Care Health Sciences, University of Oxford, United Kingdom

**Keywords:** Assistive technology, Sociomaterial, Postphenomenology, Reassurance, Older people, Chronic disease, Qualitative case study

## Abstract

The provision of reassurance is seen as a goal and benefit of the use of assistive technology (AT) in supporting people to manage their health and care needs at a distance. Conceptually, reassurance in health and care settings remains under-theorised with the benefits of experiencing reassurance through technology use assumed rather than understood. UK health and social care service goals of managing safety and risk have largely been equated with providing reassurance to users of AT and their carers. What has not been explored is how reassurance is experienced variably by users of different types of technology-enabled care.

We present data from 3 case studies of different technologies in use in health and social care provision, analysed through a postphenomenology and sociomaterial lens. Our findings point to reassurance as an important facet of AT provision but the intended functions and uses of technological devices alone did not account for people's experiences of reassurance. Participant narratives referred variously to the comfort of being monitored, having their illness/wellness verified by the device, feeling reassured by the promise of help if needed, and imbuing the device with symbolic meaning (when the user associated the device with meanings and functions other than its technical capabilities). The different ways in which reassurance was experienced provides a useful way of understanding the potential tensions with AT policy goals as well as the positive meaning attributed to devices in some cases. This study reaffirms the importance of AT implementation being anchored in what matters to the user.

## Highlights

• Reassurance is a goal of assistive technology implementation for the care of older people.
• Postphenomenology can account for variations in people's experiences of technology.
• People feel reassured through multistable human-technology relations.
• A technology's materiality can generate symbolic meaning for the user.
• Technology implementation must incorporate users' needs changing over time.

## 1 Introduction

State-sponsored assistive technology (AT) programmes have been implemented widely for their potential to provide support to people with a range of health and social care needs in their own homes (Cook et al., 2016; Steventon et al., 2013; Turner and McGee-Lennon, 2013). Focus has largely been on older people with frailty and/or complex health conditions, including dementia. However, people with physical, sensory or intellectual disabilities have also been targeted as potential beneficiaries of technology-supported care at a distance. The emphasis on distance is an important feature of how AT is promoted with many of the devices employed aiming to enable people's independence by connecting them to a system of care without necessitating in-person support. Connecting people through AT is expected to provide reassurance. There is a policy narrative that AT can reassure carers (particularly unpaid carers) through claims that AT can increase safety and reduce risk. In a survey of local authority telecare provision for older people in England (Woolham et al., 2018) 100% of local authority respondents said that one of the main ways that AT was intended to meet the needs of older people was by managing risk and promoting safety, and 81% said they viewed AT as provision to support unpaid carers. The association between monitoring a service user and the reassurance provided to carers was seen as being more important than meeting other needs such as offering information and training.

The habitual linking of assistive technologies with reassurance has been part of the policy to promote technology-enabled care services since the UK government began heavily investing in this area in the mid-2000s:

Our values are not weakened but strengthened by using these technologies to complement traditional forms of care to provide residents and their families with increased reassurance, safety, and, above all, peace of mind (Department of Health, 2005).

Reassurance is offered as a good in and of itself, available through the use of technologies rather than in relation to the risks or dangers that reassurance is required to respond to. This is an example of how assistive technology policies have adopted storylines involving seemingly logical and incontestable objectives that belie the complexity of the environment in which these technologies are implemented (Lynch et al., 2018). It is also illustrative of the technological determinism underpinning policy; instrumental in approach, these policies assume that technology behaves in predictable ways and focuses on the unidirectional effect it has on human agency (Nickelsen, 2013).

Social science studies of AT in use have considered AT as an everyday social practice (Lariviere et al., 2021), as a way of negotiating distances (Mort et al., 2003, Pols, 2012) and a part of recursive social networks that are dynamic and interactive by nature (Greenhalgh et al., 2016; Wherton et al., 2019). These studies have illuminated the work that people do (Gibson et al., 2015) and the meanings they make as they use AT, and the ways in which AT mediates relations at a distance. Technology has been understood as strengthening ties and intensifying contact between users when existing relations are already good. The experience of reassurance has yet to be examined as a sociomaterial phenomenon. We explore the concept of reassurance through empirical cases of technology-enabled care. We show how a postphenomenology and sociomaterial lens can be usefully applied to help understand variations in experiences as well as commonality between different technologies being used in different contexts.

### 1.1 Conceptualising reassurance in a health and care context

Reassurance is an under-interrogated aspect of AT research with the benefits assumed rather than understood. To reassure is ‘to restore or give confidence, peace, a sense of security, a feeling of calm, etc. to a person; to remove or allay the doubts or fears of’ (‘‘‘reassure, v.,’” 2020). Reassurance has been described as the outcome of a process and a part of processes that have as their goal relief of anxiety and restoring a sense of autonomy (Buchsbaum, 1986). Very little literature has conceptualised reassurance in the context of health and caring. Where research has considered the role of reassurance in providing care, it has been designated as a facet of the nursing profession that results in patients' decreased anxiety and enhanced ability to cope (Fareed, 1994). Studies of patients' experiences of reassurance have reported reassurance as involving empathy; receiving information and becoming knowledgeable; experiencing a tone of voice or touch which was perceived as humanistic and caring; feelings of being in a trusting relationship, physically comforting and being encouraged to be optimistic (Boyd and Munhall, 1989; Fareed, 1996; French, 1979; Teasdale, 1995). This clinically-centred framing of reassurance describes the mediating role of nurses in enabling patients to cope better with their health condition and identifies the positive potential impact of reassurance on patient outcomes.

Outside of nursing the debate centres around the usefulness (or otherwise) of invoking reassurance for patient wellbeing. Contrary to the positive qualities of reassurance indicated by the aims of AT and in the nursing literature, parent reassurance has been consistently labelled ‘distress promoting’ and potentially harmful to children undergoing medical procedures (Martin et al., 2013). In psychotherapy terms, references to reassurance are often linked to anxiety disorders and problematic behaviour known as ‘excessive reassurance seeking’. While there is no consensus on when reassurance seeking becomes ‘excessive’, emphasis is commonly placed on the persistent and repetitive nature of looking for assurance regardless of whether that assurance has already been given (Halldorsson, 2015; Joiner et al., 1999). The potentially negative consequences of the act of reassuring was noted by Gherardi (2010) in her study of an Italian telecardiology service, where reassurance was defined as: ‘A collective accomplishment achieved within a network of personal, organizational and institutional relations that mobilize people, technologies and knowledge in response to a demand – individual and collective – for the exclusion or amelioration of a justified fear.’ (Gherardi, 2010, p. 518, p. 518).

Gherardi was surprised that health professionals expressed negative views of reassurance —seeing it as an act of infantilising the patient and therefore unprofessional (Gherardi, 2010). Gherardi posited that medical professionals could view reassurance giving as distorting or minimising the problem for which the aim was to provide information and certainty. In sum, in both UK health and care policy and in scholarly literature the service-level goals of managing safety and risk have largely been equated with providing reassurance to users of technology and their carers. What is not explored is how reassurance is experienced variably by users of different types of technology-enabled care and how this provides a useful way of understanding the potential tensions with policy goals as well as the positive meaning attributed to devices in some cases.

### 1.2 Theoretical framing

Rather than viewing reassurance as something that can be ‘provided’ by the right technology, in this paper we offer a sociomaterial understanding of reassurance informed by a postphenomenological analysis. This starts with the theoretical perspective that the relationship between the social and material is constitutively entangled—not just separate spheres interacting with each other but inherently intertwined to the extent that social practices and the materiality of technologies shape each other (Leonardi, 2012; Nickelsen, 2013; Nicolini, 2006; Orlikowski, 2007). Added to this, postphenomenology brings a pragmatic framework for analysing case studies of human-technology relations, offering a way of explaining how people and technologies are connected and relate to each other in networks. Its relational ontology holds that technologies should be understood in terms of the relations human beings have with them, not as entities ‘in themselves’ (Rosenberger and Verbeek, 2015). In this way postphenomenology enables the de-centring of technological artefacts—not as objects with an impact of their own but rather as components of a system that is mutually constituted (Shaw et al., 2020). Closely allied with Actor-Network theory (ANT) it differs in its focus on the mediating role of technology in human-world relations—that is how the technology helps shape these relations—and explicit distinction between human and nonhuman entities that ANT seeks to make ‘symmetrical’, showing the continuity between actants (Latour, 1993; Rosenberger and Verbeek, 2015). Instead of symmetry, postphenomenology draws attention to the interactions and co-shaping of human experiences that occur between subjects and objects, focusing on technologies as non-neutral intermediaries between humans and the world (Rosenberger and Verbeek, 2015).

Postphenomenology offers a range of concepts for exploring different human-technology-world relations. Hermeneutic relations transform the technology user's experience of the world via direct interpretation of the technology itself (such as the read-out from scales used in heart failure monitoring or visualisation). In embodiment relations a user's experience is reshaped through the device to the extent that it may become part of the way they ordinarily experience the world—such as the consistent wearing of a pendant alarm. Alterity relations describe human interactions with technology that mimic person-to-person encounters, for example following instructions from a heart failure monitor as if they were delivered by a clinician. The notion of background relations addresses technologies that make up the user's environmental context but for the most part do not require direct interaction, for example monitoring technologies. Dissonant relations are characterised by technology being dismissed by potential users in avoidance of a magnification of unhappy relations (Shaw et al., 2020). The concept of ‘multistability’ is central to understanding how technologies are more than just ‘one thing’ (Rosenberger and Verbeek, 2015): they have multiple purposes, meanings and functions, with degrees and variations of whether (and how) they ‘work’. While a technology may be designed with a particular intended use, it cannot be reduced to *only* that usage—this is dependent on the sociomaterial context in which it functions—and equally its list of uses cannot be extended infinitely (de Boer, 2021). Stabilities are formed in relation to the perceptions of the technology user as well as to the practice or culture in which a technology is taken up. Kiran's (2015) perspective on multistability considers the ‘affordances’ of technologies—that is the socio-technical dynamics behind multiple stabilisations of devices that enable and constrain our interactions with them. Such dynamics might include the care network in which the service user is managing their needs; the trajectory of disease and interaction of multiple co-morbidities; how the technology fits into the home environment; and challenges to a person's identity from technology use. Technology in use is the result of adaptation to the material and social reality—the affordances of devices—that leads to particular stabilities (Kiran, 2012). Technologies have an actuality (how it is being used at a given moment) and a potentiality (possible future uses including unconventional or not-designed-for uses). In knowing the potential of a technology, we may take for granted that we can engage it for various types of support when we need it and without having to interact with it all the time. This ‘taken-for-grantedness’ (Kiran, 2012) shapes our actions and behaviours because we know about the technology's potential use. This perspective on human-technology interactions allows insights into the conditions in which reassurance is experienced.

Our paper does not aim to contribute to arguments about the utility of reassurance in health and care settings. Rather we intend to extend theoretical engagement with the concept by bringing a sociological perspective to provide a more critical analysis of reassurance as an embodied and interactional experience—one that involves an emotional response and in which the materiality and symbolism of the technology in use is integral to whether reassurance is perceived. We argue that reassurance is experienced through the actuality of the technology in use but also through its potentiality as a safety net in an imagined emergency situation and less obviously through the symbolic quality of devices that were often intended for a different purpose or set of practices. Symbolic qualities are those that users associated with the technology which were not explained purely by its technical capabilities (Orlikowski, 2007).

## 2 Methods and analysis

### 2.1 Situating the study empirically

The case studies we report on here formed part of a wider programme of work investigating the organisational, social, political and policy context of assistive technologies. The research took an interdisciplinary, complex systems perspective, using sociotechnical theories as an overarching framework. The wider programme initially included 6 case studies involving UK health and social care organisations embarking on new service provision supported by technology (Greenhalgh et al., 2016). Details of the case studies have been published elsewhere (Greenhalgh et al., 2017, Greenhalgh et al., 2018, Papoutsi et al., 2020, Papoutsi et al., 2020, Shaw et al., 2020) and have contributed to understanding how and in what circumstances assistive technologies are not readily adopted, used or sustained by individuals, organisations or systems. In addition to the overall findings from this programme of research about non-adoption, this paper offers an explanation of how some technologies were adopted and experienced in ways that went beyond their primary function.

### 2.2 Case studies

We focus in this paper on three case studies of technology in use for different purposes. Case study 1 investigated pendant alarms connected to a remote call centre for people with complex health and/or care needs living in the community commissioned through a healthcare organisation. Case study 2 also looked at the provision of pendant alarms but commissioned by a social care organisation to older people assessed as needing homecare or people with learning difficulties in supported living. Case study 3 investigated devices for the remote monitoring of heart failure patients. It constituted the qualitative component of a multi-centre randomised controlled trial of biomarker monitoring (weight, blood pressure, heart rate) in heart failure. Participants were given a home monitoring and communication kit (internet-enabled tablet computer and Bluetooth-enabled blood pressure and heart rate monitor, and weighing scales). Our data collection methods involved semi-structured and narrative interviews (with patients/service users, lay caregivers, health and social care staff, technology industry representatives); ethnographic visits with service users; observations of health and social care professionals’ meetings; and documentary analysis (Table 1). This paper focuses on data that relates to 19 users of pendant alarms and 28 patients trialling the biomarker monitoring kit.

**Table 1:** Methods and analytical process.

Context (3 case studies of AT)	Technology	Combined dataset	First order analysis	Second order analysis
Provision of support to older people living independently at home by 1) healthcare commissioning organisation in deprived boroughs in outer London and 2) social care organisation in mixed borough in the Midlands	Pendant alarms and base units supplied by technology companies, with alarm support services from various providers, supported by local councils.	47 index cases (end users of the technology) 82 semi-structured and narrative interviews; 61 ethnographic visits (∼80 h of observation); 20 h of observation at team meetings	Inductive analysis of dataset from case 2) identified reassurance as an important theme Followed by: Deductive analysis of cases 1 and 3 identified further data on reassurance	Analysis of the relations between people/tech/world in terms of: hermeneutic relations, embodiment relations, alterity relations, background relations and dissonant relations
3) Remote biomarker monitoring in heart failure patients living at home by acute hospital trusts in six different cities in UK	Tablet computer and Bluetooth-enabled commercially available sensing devices (blood pressure and heart rate monitor, weighing scales)			

Ethical approval was obtained for each case study from University of Birmingham Research Ethics Committee (ERN_11–0598); Oxfordshire South Central Research Ethics Committee (REC no. 15/SC/0553) and NRES Committee London: Camden & Islington Research Ethics Committee (Ref 13/LO/1610).

The study team held cross-case meetings between 2015 and 2020 to analyse the case studies both individually and across cases in a process of synthesising findings and identifying commonalities and contrasts. Through this process an emerging theme of ‘reassurance’ was identified as a common feature across 3 case studies notwithstanding the different technologies and contexts involved. We developed the research questions: how does reassurance emerge through human-technology interactions? What conditions need to be in place for reassurance to be experienced? And who are the expected beneficiaries? The methods and data we used to address these questions are summarised in Table 1 below.

To analyse the human-technology interactions of interest, we turned to postphenomenology as our primary analytic lens. We applied the postphenomenological concepts of hermeneutic relations, embodiment relations, alterity relations, background relations, and multistability to our empirical data (Ihde, 1990, 1993), comparing these relations across our cases. Together, these concepts allowed us to theorise about how reassurance was produced by assistive technology use in our case studies illuminating how different technological devices assumed meaning and acted in multiple ways including to reassure their users.

### 2.3 Findings

The ways in which reassurance emerged through technology use in our cases highlighted the multistable and context-dependent human-technology relations. Pendant alarms and heart monitoring devices mediated relations in different ways—enabling and constraining the user's behaviour and actions due to their respective affordances. This variation resulted in different reported experiences of reassurance. There were obvious distinctions between the two technologies: they differed in their primary intended functions, material properties, their users (and networks of care) and their everyday use. The pendant alarm was designed to support older people to live independently at home and to be used in specific, irregular circumstances. It was nonactivated for the majority of the time but provided an important communication line between the user and a call centre in cases when the user needed third party assistance, such as having fallen or experiencing anxiety. In this way the pendant alarm was used in response to an emergency situation as framed by the user when they made an active decision to press the button and call for help. Conversely, the heart failure package was intended to provide regular monitoring of a specific chronic health condition to support medication decisions and patient self-management. It required the commitment of daily input—use of a tablet computer to complete a symptom checker and then two separate devices (blood pressure/heart rate monitor and weighing scales) to take measurements. The heart failure monitoring device connected patients with a health care team through the automatic transfer of data, which was obtained by patients and combined with other available information (such as that from electronic health records) to support clinical decisions to alter patient medication. The different intended functions and uses of the devices alone did not account for people's experiences of reassurance. Participant narratives referred variously to the comfort of being monitored, having their illness/wellness verified by the device, feeling reassured by the promise of help if needed, and imbuing the device with symbolic meaning (when the user associated the device with meanings and functions other than its technical capabilities). We examine these ways in which reassurance emerges from technology in use below, before describing examples of devices causing distress or negative feelings about the role of technology in care.

#### 2.3.1 Reassurance from human-technology relations

A focus on the different forms of technological mediation revealed the dominance of the hermeneutic relation between the user and the technology in the heart failure case, with the focus often being on the technology readout. Judith reflected on how the heart failure devices provided verifying information that supported her illness experience.

Judith: I still weigh myself every day. I don't - I could take my blood pressure every day, because I've got a blood pressure machine. But I don't bother. It's not worth it. You know? But sometimes if I feel a bit strange, if I feel my heart's a bit funny, [um] or - which, as I say - touch wood - is not too bad at the moment - [um] I would go and take my blood pressure, just to [er] see where it is. But [um] it was strange. Because then it became - It sort of [um] - Then it was a bit of a crutch.

I: Yeah. Tell me a bit more about that? Sort of how did it?

Judith: Well, because you felt there was somebody monitoring how well or not well you were, on a continuous basis. Which was a comfort.

Judith made a connection between how she felt and what she perceived to be objective information about her health status. Such patients were able to assume a central role in managing their condition, experiencing reassurance through a sense of being informed and in control. For one participant, Susan, the monitoring device gave her vindication and confidence that the symptoms she was experiencing had an explanation. Susan spoke of not being believed by medical practitioners and her worries about being abandoned by the health service following an operation:

Susan: Because a lot of people walk around with things and they're not quite sure. It's like me with thinking ‘there is something there’, but - and then being told by your medical people ‘well there's nothing wrong with you'.

Interviewer: Yeah. Yeah.

Susan: And you think ‘well, there is, and maybe I'm going paranoid, but there is something, I know my own body and there is something'.

I: It [the monitor] may help identify things.

Susan: Yeah. And it might be useful.

I: Okay.

Other participants talked about the importance of seeing the data in graphs (so they could understand patterns in the readings) and being able to use ‘a proper decent bit of kit’ (heart failure study participant, Jim) rather than just jotting numbers down on pieces of paper. A key aspect of the hermeneutic relation is the extent to which the technology user knows how to read the technology and the various ways in which the heart failure data were presented to patients seemed to reinforce participants' knowledge of their condition. In this way the technology itself was not responsible for the reassurance experienced, rather how individuals related to their devices was key.

In some instances, the readout from the monitoring equipment took on a ‘quasi-other’ quality (Ihde, 1990). Heart failure study participants Michael and Judith took instruction from the monitoring system, responding to the assurances from the readout as if they were coming from a clinician:

Michael: My wife liked it a lot, actually. I mean, you mentioned my wife - She gets in an awful flap about my health. And it was - She just liked knowing. Particularly with blood pressure and weight, that it was okay. And she - You know, I - She, she could ask me if it was okay. And I - I'm not an expert on these things, but I got to know what was - you know, when blood pressure was high or low. Because what I liked about the system was you know, if you did it and it was high, it sent back a message saying ‘your blood pressure is higher than normal’, or … Something like that. ‘You may want to ring your doctor'.

–

Judith: And as I say, that was - That was good, to [er] - to have somebody there. Or okay, it's only a machine, but it was something that sort of ‘oh, that's alright then, I'm okay today’. [laughing].

These examples of alterity relations show how patients sometimes related to their devices in ways that could be deemed similar to how they would interact with other humans, which is not to suggest the devices were mistaken for humans but rather that they were happily accepted as representations of the clinical team monitoring their data and thus felt reassured by these interactions.

Many of the examples from our heart failure study described how the technology afforded connection to a system of support. Richard was 76 years old, single and living in shared accommodation:

Richard: I knew I were being kept an eye on, [laughing] by more than one or two people.

I: And by one or two people, who do you mean? Who do you mean was looking after you?

Richard: Well, not just my doctor. But the hospital […] And yourselves [meaning the clinical trial team]. Yeah. They'd got their eye on me. [laughing].

For some, the resulting reassurance was linked to the anxiety they lived with as part of managing their disease and how they felt the monitoring devices helped to alleviate this. Dennis, a man in his seventies who lived with his wife, talked about this during his interview:

Dennis: […] I think for people with heart problems, you tend to panic. […] I would like to put up my hand and say ‘I never do’. But I - you know, you do worry. Worry is a better word than panic, I think. And this programme, with - you know - providing me with the kit, was actually a great reassurance.

Such insights into the daily worries of people's lives demonstrate how the requisite conditions for reassurance to be sought and given were created. In the context of heart failure we also heard how the type of reassurance and support needed may change during the course of the condition, illustrating the multistability of human-technology relations. Judith found the technology reassuring when she was first diagnosed but later expressed not needing it in the same way. For her it was potentially reassuring not to have to use the technology at all:

Interviewer: So, would you - If you had the opportunity to continue using it then, would it be something that you'd do, or?

Judith: [sigh] I don't - I would do it if it was - I wouldn't want to have to do it indefinitely.

I: Yeah. Mmm.

Judith: I don't think. Because I'm lucky, that I've improved and [um], you know, I've got used to the idea of ‘heart failure’ [spoken in a gloomy voice].

I: [laugh] Yeah. I can see what you mean. So you don't feel like you need to monitor yourself all the time. Yeah.

Judith: No. No. I don't feel quite so vulnerable. Because I did feel vulnerable when I first had it happen.

The technology-mediated connection to a support system became less meaningful as the socio-technical dynamics changed.

Reassurance from technology potentiality.

In considering technologies' affordances we found examples in our data of participants engaging with the *potentiality* and not just the *actuality* of their devices (Kiran, 2015)—namely, describing their reassurance from knowing that help would be there if they needed it. This was different to an expectation that using the technology at any given moment (actuality) would trigger help as in an emergency situation; rather it was the imagined scenario of the technology as a safety net (potentiality) that provided users with reassurance that in the event of a sudden change in their health or wellbeing (e.g. a fall or unusually high blood pressure reading) the technology would mediate an appropriate third-party response (e.g. a visit or phone call from a health/care professional). The pendant alarm provides a useful example of a technology that is permanently plugged in and connected to a call centre but remains nonactivated most of the time. As with other technologies in the user's environmental context, the user may share background relations with the pendant alarm—rarely interacting with it directly but nevertheless making choices and experiencing the world in light of its presence. Zainub, a South Asian woman in her eighties, who lived alone but had regular contact with her children and grandchildren, referred to the potentiality of her device. She felt reassured that she could press the button to the call centre and get a helpful response if she needed it.

Interviewer: And do you manage okay?

Zainub: Yes.

I: Do you do all of that yourself?

Zainub: Yes.

I: Yeah.

Zainub: I can't depend on anybody. I don't think anybody's got time these days, unless I have community service.

I: Do you worry about that?

Zainub: No.

I: You think you're okay to manage?

Zainub: So far, with God's help, I'm alright.

I: Yeah.

Zainub: But, er, the future, God knows, we'll see. But people are very wonderful. I mean, I just have to press this [pendant alarm button] and they call me if I want any company, but I don't want to trouble anybody. But they're very polite, they're very nice.

Participants in the heart failure study had a different level of interaction with their devices, which required them to input daily measurements and answer questions on the tablet computer. Nevertheless, we identified a difference between the reassurance they experienced from the immediate response (readout) that showed, for example, a continuity or improvement in their measurements, and the common expectation that the technology readout would mediate contact from a clinician if necessary:

Dennis: Well, one thing I thought was excellent, when I was on the research programme, was every day I took my blood pressure. Was one of the three things I was doing. And if there was a major change, then [a nurse] or somebody rang up, to see I was alright. And you know, the same with weight.

Interviewer: Mmm.

Dennis: I weighed myself every morning, just inputted [my symptoms] into the iPad.

I: Yes.

Dennis: I was very impressed with that, actually. It gives you a lot of reassurance.

I: Right.

Dennis: And if there was anything really wrong, I would - you know, I could ring them up and presumably they would do something. But nothing ever was. [laughing].

–

Interviewer: So, what was it that gave you the reassurance that someone was looking after you, in terms of the data you were sending?

Richard: Well, any problem - you know - and it was going, could be highlighted and somebody would dive on it.

In these examples it is the expectation of someone responding that is important, rather than the action of responding. The reassurance that is achieved comes from knowing help is there *if* needed rather than as an immediate consequence of using the AT.

#### 2.3.2 Reassurance through symbolic meaning

One of our participants described a relationship with his pendant alarm that was different to those defined by a connection to a wider system of support or expectation that help would come when needed. Arthur, a man in his eighties, had lived alone since his parents died. He had never married or had children and his narrative was coloured by a sense of being wholly responsible for his wellbeing with increasing concerns about becoming less able to cope with everyday life.

Arthur: So I've been on my own 40 years, and on my own, having to do everything myself. Now, it's like this. See that cup? That'll stop there ‘til I move it. And the same with everything. The garden won't get done on its own, unless I do it. The house, well, yeah, it could be cleaner, but unless I do it … And so you're conscious of this. And as you get older, you know damn well [it's not getting easier]. Now, I've worked all my life, unsocial hours, and my body is [like] a car, I'm wearing out. At times, I feel worn out. And so when you're on your own, you think … I mean, as I was saying to Thelma yesterday, she said ‘I don't see anybody’. I said, no, I said, but Thelma, you're a bit different, you've got kids and grandkids. And they're fairly good. But when you're on your own … you're more conscious of it.

Arthur saw the community alarm installed in his house as a lifeline that provided him with continual reassurance. He had in the past fallen in the garden when he wasn't wearing the pendant and since then wore it around his neck at all times. In this way, Arthur's relationship with his pendant alarm was embodied, reshaping the way he experienced the world. He found the object reassuring even when he chose not to use it, but also when he knew it *could not* be used. Arthur explained in an interview that despite a number of incidents where he had become suddenly ill, he had never resorted to pressing the alarm:

Interviewer: But even on that day when you were up at one o'clock in the morning, struggling to breathe, you didn't press it?

Arthur: No, because I'd had it before and I've raced out of the house before … But I got my breath. But it gives you that reassurance.

In everyday use, the pendant alarm was pivotal in making a connection between Arthur and the support network available via the call centre. Whilst he chose not to press the alarm, its presence reassured him that he could if needed. However, Arthur also found reassurance in the physical presence of the alarm even when he knew that it would not connect him to his support network; he wore the alarm when he was away from home at football matches as he describes in the interview extract below.

Arthur: Wherever I go, I don't take it off me. If I go into [town] or I go down to London regular, it's always round my neck. It's no good but at least I've always got it round my neck. And when you're on your own, things feel always far worse than they really are. I can always press it. And that's reassuring … But if I collapsed, what else do they use [for identification]? I've got this round my neck. This tells people, because they can check up with that. Although it's no good effectively where you are, you've got your [pendant] and so you've got the reassurance.

Arthur's wearing of his pendant alarm is routine and embedded into his everyday life. Because he has it at all times he knows tacitly that he will always have it—there is no cognitive demand of knowing when to put it on or take it off. Arthur rationalises his wearing of the pendant out of range (i.e. when he knows it can't function as it was designed to) to an extent by pointing out that it acts as a sign to other people that he might need help:

Arthur: You hear the thing, older - well, I'm older - vulnerable person. That's a theme, older, vulnerable person. You hear it thrown about in local and national government. And it's true.

Wearing the pendant alarm identifies Arthur as an ‘older, vulnerable person’, part of a group of people that (he feels) are looked after in government policy. Even when he is unable to use it to summon help in its conventional sense, Arthur seems to believe that the pendant alarm can signal to others that he might need help. In wearing the pendant alarm for reassurance whilst knowing it can't function in the way intended, Arthur imbued the technology with a meaning that was related to, but not contingent on, its functional purpose. The pendant alarm, when working in this way, became more than an inanimate object containing a button to be pressed, acting to symbolically connect Arthur with other people. The sense of reassurance Arthur felt relied on the physical, material presence of the alarm; we would not have expected Arthur to be reassured by his pendant alarm whilst he was out and about if he had left it at home. Here we can see how the social (Arthur's connections with supportive networks) becomes ‘constitutively entangled’ (Orlikowski, 2007) with the material (the comforting physical presences of the pendant). The pendant alarm holds amuletic properties for Arthur. Amulets are understood to have power for the wearer (The Pitt Rivers Museum, 2012), offering them protection. Arthur's amuletic use of his pendant alarm provides him with the comfort of its physical presence as a constant reminder that he possesses an object through which he could summon help. The pendant alarm worked for Arthur by mediating his relations with the world through a set of beliefs and experiences that related to but were not wholly explained by the capabilities of the technology.

The pendant alarm can be understood as working for Arthur by connecting him (metaphorically and symbolically as well as materially through remote transmission) with people who he believes will help him. Wearing the pendant alarm reassured Arthur by signifying his connection, and his need for connection, with other people who he expects to help him.

#### 2.3.3 Adverse experiences

Some users experienced negative interactions with technology, including anxiety and resentment. For example, Harry, a man in his sixties with learning difficulties and comorbid health conditions showed confusion about his pendant alarm package. The community alarm ‘hub’ that sat alongside his television and to which his pendant alarm and other sensor devices (e.g. a wrist-worn falls detector) were connected caused him distress, as he describes in the interview below:

Harry: I mean, it seems as if it keeps going off every so often.

Interviewer: How does it go off, is it an alarm?

Harry: I don't know if it's an alarm or what when it goes off. Because I know when I press that, er … the whatsaname in the middle of that watch type thing …

I: Oh, yeah, the falls watch. Yeah.

Harry: That, er … that sets it off at times when I've used that, and got through to the people I've got to talk to.

I: Yeah. So do you mean that when it goes off, it goes off when you don't want it to?

Harry: It makes me wonder what's caused it to go off.

I: Yeah. And does that worry you?

Harry: Making me wonder if they're trying to get in touch for any reason.

I: And do they try and call you through that [community alarm box]?

Harry: I'm not sure.

Harry did not understand the workings of the community alarm or why, when he pressed its button, someone would try to talk to him through the white box by his television. Rather than offering Harry a connection, the technology offered a hermeneutic relation which made no sense, provoking anxiety rather than reassurance.

Gordon, an older man with multiple chronic health conditions, talked about the issuing of his community alarm device as indicative of the council's consistent failure to meet his needs:

Gordon: The council have always let me down in not getting the help that's needed […] What annoys me most of all, they put stuff on that I'm not really interested in, and they talk a lot of rubbish, a lot of them. I'm not interested in that either.

Gordon's relationship with technology was dissonant: the introduction of technology magnified the unhappy relationship between Gordon and the council which had ‘let him down’, leading to rejection of the technology.

The experiences of Harry and Gordon further demonstrate the multistability of these devices and the implications for reassurance, which cannot be achieved if the technology is mismatched to the potential user's needs. The purpose of the pendant alarm is to connect users to support, usually in an emergency situation. In these two cases insufficient attention had been paid to the sociomaterial contexts in which these devices were installed and the meaning the users would attach to the technology. For Harry the technology was unusable—he lacked the capacity to engage with it as it had been intended and its presence caused him distress. For Gordon the dissonant relation provoked active rejection of the technology—his past experience contextualised his perception that the device exacerbated rather than alleviated the challenges he faced.

Our case studies illustrate the different relations people had with their technological devices and how these resulted in feelings of reassurance. For some participants reassurance was directly related to the primary function of the AT device—i.e. engagement with the data readout from the heart failure monitoring kit. We have also presented examples where reassurance came not from direct interaction with the AT but from a belief in its potentiality and trust in a wider support system that would mobilize as needed. In addition, we have described how a technological device can be imbued with symbolic power that is tied to its material presence and not wholly its functionality. Whether reassurance is experienced is dependent on the context in which the AT is implemented.

## 3 Discussion

We began this paper by noting the conflation of the assistive technology policy goals of managing safety and risk with providing reassurance to users of the technologies. Our study has found that what is promoted as being reassuring and what is experienced as reassurance can be different. A postphenomenological lens has enabled us to illuminate the different human-technology relations that shape users' experiences of AT and accounts of reassurance. We have identified how multistability affects users' interpretation of technologies and therefore their use, adaptation and rejection of them. For some of our participants this manifested as adverse experiences due to their individual requirements being disregarded. This reflects recent research findings of inadequate assessment processes for AT that show the disconnect between the focus of services providing AT and the desires of service users (Forsyth et al., 2019; Woolham et al., 2021). A key element of Gherardi's (2010) definition of reassurance is the presence of a justified fear or anxiety providing the impetus for reassurance seeking and giving. While we did not seek to assess the justification of our participants' fears, it was clear that many experienced the burden of worrying about their health and ability to self-manage, and some—like Arthur—expressed feelings of disconnection. For these people, technology connected them with a support network and provided reassuring readouts.

Reassurance was felt through different technology-mediated relations as the fears and anxieties that prompted the need for reassurance changed, re-emerged or continued. Different forms of technological mediation worked in varying ways to help users feel in control by reinforcing their knowledge about their condition and connecting them to supportive others. This only worked, however, if the technology and the people it connected them to were meaningful to the user in terms of meeting a need they had identified, and that they still needed to be met. For some, such as heart failure patient Judith, that need was apparent at one stage of their disease trajectory but not at another. The anxiety that this participant had felt early on dissipated over time. López and Domènech (2008) have theorised the tension inherent in people's self-care strategies when engaging with technology—sometimes seeing themselves through the lens of a ‘body-at-risk’ that requires constant surveillance; and other times invoking the ‘vigorous body’ that enables daily activities but is pushed to the background in a way that denies its problematic nature. In the latter scenario, the user may not comply with expectations about how to use the technology that is supporting their care if to do so raises questions about their body or destabilises their way of life (López and Domènech, 2008). Thus, a heart failure patient who has adjusted to recent diagnosis may no longer identify with the vulnerability narrative attached to their technological device and may be unwilling to imagine a future emergency scenario that could be mitigated by continuous AT use so will stop using it.

The question of whether reassurance is a helpful experience to seek was not one that arose in our study. The benefits of reassurance (and the potential for technology to contribute towards reassurance) were assumed, unlike some of the literature which frames reassurance as potentially having negative consequences. In Gherardi's study of telecardiology, health professionals questioned the need for reassurance when the basis for the technology-enabled service was to enhance their knowledge and ‘search for certainty’ (Gherardi, 2010: 517). For them, the idea of reassurance undermined their intention to provide concrete information. In our study we saw examples of reassurance that could be viewed as juxtaposed to the search for certainty. Participants who were reassured by the potentiality of a safety net (rather than the actuality of one) were not searching for the certainty of it—imagining a safe future was enough. The outcome of reassurance from the symbolic use of a technology (as when Arthur wore a pendant alarm out of range) was produced not from the knowledge that he was safely connected to a network of support but from a sense of security in spite of such knowledge. Reinforcing knowledge was just one aspect of the multiple ways reassurance was felt. Taking a postphenomenological perspective demonstrates how AT such as the heart failure package affords constant self-surveillance, shaping people's experiences of health and illness by blurring the distinction and invoking the body-at-risk (Hofmann and Svenaeus, 2018; López and Domènech, 2008). In this context, there is an inevitability about the search for reassurance.

A limitation of our study is that we did not seek the perspectives on reassurance of family members or health and social care professionals. The focus in AT policy on the potential to reduce burden on carers and services has been well documented (Davies et al., 2013; Gibson et al., 2015; Steils et al., 2021). However, to fully answer the question of ‘who benefits from reassurance?’ the role of technologies in affording family members and health and care professionals reassurance needs to be considered alongside further exploration of carers and professionals as part of these socio-technical arrangements. The reassurance experienced by technology users in our cases, through multistable relations and instilling symbolic meaning, was an important part of navigating risks and feeling safe. The outcome is not necessarily a perceptible change to the technology user's situation but it may give the individual confidence to live more as they would like, with the unavoidable risks associated with their condition. At a fundamental level, the presence of reassurance may stabilise existing care arrangements, keeping under control everyday relationships of dependency (López and Domènech, 2008). A more critical reading of the collective work that produces reassurance through symbolic properties of technology is that care arrangements which are not optimal are perpetuated, offering reassurance instead of safety. Health professionals in particular have, in the past, reported safety concerns about remote monitoring of patients with long-term conditions in relation to technical issues compromising the accuracy of clinical assessments and the increase of data leading to over-treatment and side effects (Brunton et al., 2015). The potential for false assurance has been identified in telehealth research, for example, when patients misunderstand the scope of the monitoring technology and remit of the clinicians in the socio-technical network leading to possible delays in help seeking (Brunton et al., 2015; Ure et al., 2011). Nevertheless, this does not mean that the patients in our study who described a false sense of security were at risk as we found that most health professionals only registered their patients in the trial when they were deemed stable enough not to need urgent care (Authors, 2020).

## 4 Conclusion

The concept of reassurance in an AT context has received little scholarly attention (despite it being a sought-after effect by care providers and technology manufacturers) but our findings show it to be an important factor in people's relationships with the devices intended to support them. We have drawn on sociomaterial and postphenomenology perspectives to demonstrate how reassurance is a subjective experience felt by technology users and their supporters in particular contexts and as a result of different concerns. Rather than there being evidence that individual devices enable or inhibit reassurance, it is the multistability of the technology and the configuration of people, objects and context that produces different modes of reassurance. There are conditions which are more or less conducive to enabling experiences of reassurance. In some circumstances the attempts to establish these conditions produce adverse experiences leading to dissonance and alienation. The framework of concepts provided by postphenomenology has helped us to account for the variations in people's experiences and has equally highlighted the commonalities in different types of technologies being used in different circumstances.

We have explored and extended Gherardi's theory of reassurance as a social practice, augmenting an understanding of the practical accomplishment of reassurance through a broader appreciation of the different contexts in which this may be situated. We have also emphasised the importance of the technology's materiality in producing this effect. Whilst we acknowledge that materiality is not synonymous with physicality (Leonardi, 2012), it is clear that for some people in some cases the physical presence of the technology generates significant symbolic meaning. This highlights the importance of technology implementation being anchored in what matters to potential users, employing co-design principles in the manufacturing of technologies, and understanding how needs may change over time (Gray, 2020; Greenhalgh et al., 2015). Furthermore, this needs to be supported by a policy agenda that recognises the socio-technical complexities that govern the success of technology implementation (Eccles, 2021).

## Credit author statement

Jennifer Lynch: Conceptualization, Methodology, Investigation, Data Curation, Writing – Original Draft. Gemma Hughes: Conceptualization, Methodology, Investigation, Writing – Original Draft. Chrysanthi Papoutsi: Investigation, Data Curation, Writing – Review & Editing, Joseph Wherton: Conceptualization, Investigation, Writing – Review & Editing. Christine A'Court: Methodology, Investigation, Writing – Review & Editing.
